# The diet–intestinal microbiota dynamics and adaptation in an elevational migration bird, the Himalayan bluetail (*Tarsiger rufilatus*)

**DOI:** 10.1002/ece3.11617

**Published:** 2024-06-29

**Authors:** Shangmingyu Zhang, Chuang Zhou, Zhehan Dong, Kaize Feng, Kexin Peng, Zhengyang Wang, Yong Jiang, Linyu Jin, Ping Zhang, Yongjie Wu

**Affiliations:** ^1^ Key Laboratory of Bio‐resources and Eco‐environment of Ministry of Education, College of Life Sciences Sichuan University Chengdu China; ^2^ Department of Organismic and Evolutionary Biology Harvard University Cambridge Massachusetts USA; ^3^ Administration of Gongga Mountain National Nature Reserve Kangding Ganzi Tibetan Autonomous Prefecture China; ^4^ Chengdu Tianfu International Airport Branch of Sichuan Airport Group Limited Company Chengdu China

**Keywords:** dietary habit, DNA metabarcoding, elevational adaptation, elevational migration, Himalayan Bluetail, intestinal microbiota

## Abstract

Migratory birds experience changes in their environment and diet during seasonal migrations, thus requiring interactions between diet and gut microbes. Understanding the co‐evolution of the host and gut microbiota is critical for elucidating the rapid adaptations of avian gut microbiota. However, dynamics of gut microbial adaptations concerning elevational migratory behavior, which is prevalent but understudied in montane birds remain poorly understood. We focused on the Himalayan bluetail (*Tarsiger rufilatus*) in the montane forests of Mt. Gongga to understand the diet–gut microbial adaptations of elevational migratory birds. Our findings indicate that elevational migratory movements can rapidly alter gut microbial composition and function within a month. There was a significant interaction between an animal‐based diet and gut microbiota across migration stages, underscoring the importance of diet in shaping microbial communities. Furthermore, the gut microbial composition of *T. rufilatus* may be potentially altered by high‐altitude acclimatization. An increase in fatty acid and amino acid metabolism was observed in response to low temperatures and limited resources, resulting in enhanced energy extraction and nutrient utilization. Moreover, microbial communities in distinct gut segments varied in relative abundance and responses to environmental changes. While the bird jejunum exhibited greater susceptibility to food and environmental fluctuations, there was no significant difference in metabolic capacity among gut segments. This study provides initial evidence of rapid diet–gut microbial changes in distinct gut segments of elevational migratory birds and highlights the importance of seasonal sample collection. Our findings provide a deeper understanding of the unique high‐altitude adaptation patterns of the gut microbiota for montane elevational migratory birds.

## Graphical abstract

We conducted the first investigation into the seasonal dynamics of diet–gut interactions within Himalayan bluetail (*Tarsiger rufilatus*), a migratory songbird known for its elevational movements. This species experiences year‐round elevational shifts and endures drastic environmental variations such as hypoxia, hypothermia, and limited food availability. This research provides initial evidence of rapid diet–gut microbial changes in distinct gut segments of elevational migratory birds.

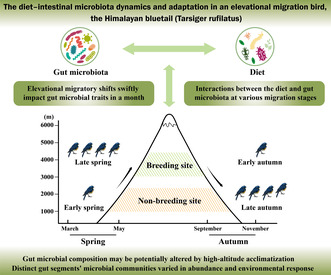

## 1 INTRODUCTION

The gut microbiome is widely recognized as the “second genome” of animals (Weinstock, 2012). It has coexisted and co‐evolved with its host animal, influenced by genetics, diet, behavioral habits, and various intrinsic or extrinsic factors (Ruiz‐Rodriguez et al., 2018). Birds, known for their high dispersal capacity, have complex life history strategies in response to seasonality and fluctuating environments. In this context, the co‐evolving gut microbiota assumes a pivotal role in facilitating seasonal environmental adaptation (Grond et al., 2018). Understanding the natural co‐evolution of the host and gut microbiota is critical for elucidating the diversity and environmental adaptation of the avian gut microbiota.

Migratory birds possess complex and diverse gut microbiomes due to their seasonal and extensive movements (Zhang et al., 2021). Recent studies have revealed the influence of migration and environmental factors on the interaction between diet and gut microbiota (Grond et al., 2014; Ryu et al., 2014; Skeen et al., 2023). Migratory birds face physiological challenges during long‐distance movements, including energy demands, environmental stress, and immunity, which result in weight loss and gastrointestinal atrophy (Buehler & Piersma, 2008; McWilliams & Karasov, 2001; Weber, 2009). These extreme physiological challenges affect host–microbial interactions and lead to variation in gut microbial communities (Grond et al., 2019). Understanding the formation and maintenance of microbiota associated with extreme physiological challenges in migratory birds and exploring their effects on host adaptations are important for comprehending avian physiological adaptation. Despite this, the phenomenon of elevational migration, exhibited by around 10% of the world's approximately 10,000 bird species (Barçante et al., 2017), remains largely unexplored in relation to the avian gut microbes.

Elevational migratory behavior is a common yet understudied trait in montane birds, involving seasonal round‐trips across changing altitudes (Boyle, 2017; Hsiung et al. 2018). Large changes in elevation correspond to drastic alterations in environmental factors, including oxygen partial pressure, air density, temperature, and ultraviolet exposure. Therefore, the physiological challenges for high‐altitude migratory birds differ from long‐distance migrators. While the latter prioritize efficient energy management, high‐altitude migrators focus on rapid physiological adaptations, altering behavior, feeding strategies, and metabolic capacity (Williamson & Witt, 2021). The gut microbiota of birds is closely intertwined with diet, behavior, social contact, and external environmental factors, exerting substantial influence on digestion and absorption, immune regulation, and vitamin synthesis (Capunitan et al., 2020; Ley et al., 2008). Therefore, investigating the effects of movement on gut microbial dynamics in elevational migratory montane birds provides insights into host‐microbe relationships in the context of rapidly changing environments and recurring physiological stresses.

As a representative montane endemic passerine bird, the Himalayan Bluetail (*Tarsiger rufilatus*) weighs 12–16 g and exhibits seasonal elevational migration behavior and sexual dichromatism (Morimoto et al., 2006). Previously considered a southwestern subspecies of *T. cyanurus*, *T. rufilatus* has recently been recognized as a distinct species (Luo et al., 2014). Unlike long‐distance migratory *T. cyanurus*, *T. rufilatus* breeds in the Hengduan Mountains and demonstrates elevational migratory behavior. During the breeding season (typically March–May), they migrate to high‐altitude sites above 3000 m. In the non‐breeding season (usually October–November), they descend to lower altitudes below 2000 m (DuBay et al., 2020; Williamson & Witt, 2021). Thus, this species experiences varying environmental pressures throughout the year, even within the same month. This provides an excellent opportunity to investigate cyclic variations in diet‐gut microbiota dynamics and adaptation.

In this study, we investigated gut microbiota variations across different intestinal segments during four migration stages of *T. rufilatus*: early spring (March–April), late spring (April–May), early autumn (October), and late autumn (November). Additionally, we analyzed the dynamics of diet–gut microbiota interactions in different gut segments to understand the impact of elevational migratory behavior. These insights contribute to a deeper understanding of the distinctive adaptive patterns of gut microbiota in montane birds during high‐altitude migrations.

## 2 MATERIALS AND METHODS

### 2.1 Study site and sampling

**FIGURE 1:**
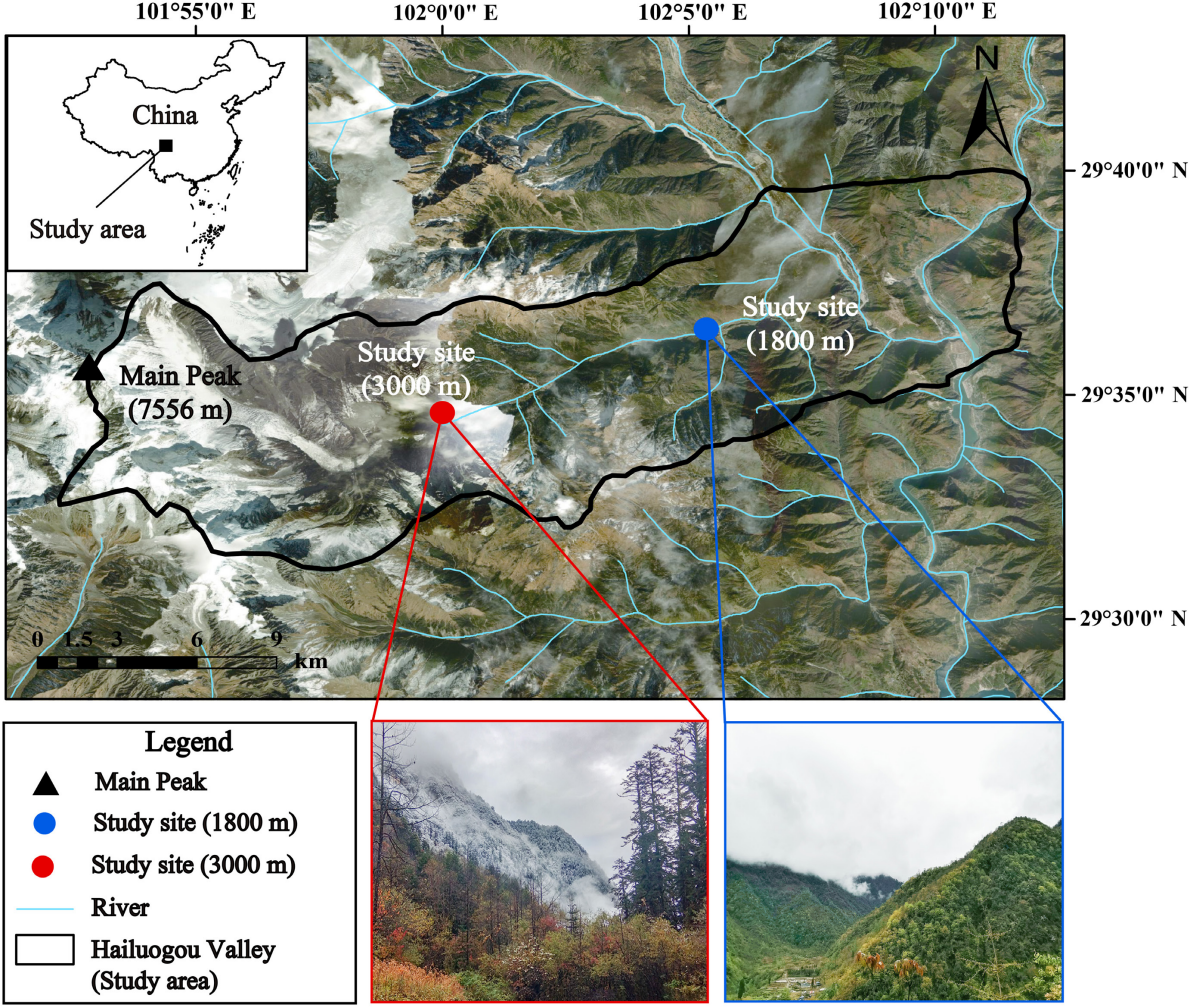
Sampling sites and habitat photos.

We captured *Tarsiger rufilatus* (*n* = 22) during two migration seasons: the spring migration period (March 14–May 7, 2021, *n* = 11) and the autumn migration period (October 12–November 2, 2021, *n* = 11) in Hailuogou Valley of Mt. Gongga (29.576096° N, 101.998624° E) on the eastern edge of the Tibetan Plateau (Figure 1). In this region, *T. rufilatus* migrates upslope to an elevation of 3000 m during the breeding season and migrates downslope below 2000 m in the non‐breeding season (Figure S1). Thus, we set up 11–13 mist nets (12 m long) at two elevational bands (1800 and 3000 m) to sample this species simultaneously. The mist nets were opened from sunrise (±1 h) to sunset (±1 h), during suitable weather conditions. All captured individuals, determined to be adults based on plumage assessment and confirmed to be at least 1‐year old, are detailed in Table S1 with specific information. Fresh feces were collected within 15 min of individual capture, followed by immediate euthanasia by cervical dislocation, and the contents of the stomach, duodenum, jejunum, and ileum were extracted sequentially according to anatomical structure (Figure S2). The contents of each were stored separately in liquid nitrogen after collection and then transported to Sichuan University for further processing. We also recorded temperature and humidity at each sampling site using a data logger (Kongsaien, COS‐04), which logged the maximum and minimum temperature for the day of capture for each individual.

All animal research procedures complied with the regulations of the Gongga Mountain National Nature Reserve and the Animal Care Review Committee in the College of Life Sciences, Sichuan University (No. 20201208001).

### 2.2 Stomach content processing

The gastric contents were freeze‐dried using a vacuum freeze‐dryer and then examined under a stereomicroscope (Nikon SMZ 745T) to assess similarity based on undigested food fragments. Samples were categorized into four groups, representing low/high altitudes in both spring and autumn, with each group comprising three samples. Each sample was a composite of stomach contents from three different individuals, resulting in a total of 12 samples. Detailed sample information is provided in Table S2. This ensured adequate DNA volume for metabarcoding analysis, with at least one individual per pool analyzed for gut microbiota. For DNA extraction, we employed Fast DNA SPIN extraction kits (MP Biomedicals, USA). The extracted DNA was quantified using the NanoDrop‐1000 Ultra‐Micro Spectrophotometer (Thermo Scientific, USA). To monitor for any potential contamination, standard negative controls were included in the DNA extraction process. Any signs of contamination, such as unexpected DNA bands in gel electrophoresis, abnormal readings in the NanoDrop spectrophotometer, or the presence of non‐target DNA sequences detected by PCR, observed in the negative controls led to a thorough review and repetition of the extraction process for the affected samples to ensure the integrity of our data.

### 2.3 Diet DNA metabarcoding and analysis

To analyze animal‐based food, we utilized primers ZBJ‐ArtF1c and ZBJ‐ArtR2c to amplify the mitochondrial COI gene fragment (Table S3). For plant‐based food content analysis, we employed primers ITS2‐F and ITS2‐R to amplify the internal spacer transcribed region ITS2 fragment of ribosomal DNA (Table S3). We implemented standard negative controls to prevent contamination and conducted preliminary tests to validate the PCR reactions' specificity and efficiency, obviating the need for explicit positive controls. In the PCR assays, we mixed 5 μL of Q5 reaction buffer (5×), 5 μL of Q5 high‐fidelity GC buffer (5×), 0.25 μL of Q5 high‐fidelity DNA polymerase (5 U/μL), 2 μL of dNTPs (2.5 mM), 1 μL each of forward and reverse primers (10 uM), 2 μL of DNA template, and 8.75 μL of ddH_2_O. The PCR amplification parameters included pre‐denaturation at 98°C for 2 min, denaturation at 98°C for 15 s, annealing at 55°C for 30 s, extension at 72°C for 30 s for 25 cycles, and a final extension at 72°C for 5 min. PCR products were purified using Agencourt AMPure Beads (Beckman Coulter, IN) and quantified with PicoGreen dsDNA (Invitrogen, USA). We opted not to replicate the same sample for sequencing, relying on our extensive sample collection and rigorous quality control from DNA extraction to sequencing to ensure the reliability of our results without compromising sample diversity. The DNA library was prepared using the TruSeq Nano DNA LT Library Prep Kit for Illumina. The ITS2 fragment was sequenced using the MiSeq Reagent Kit V3 and Illumina MiSeq 2500, generating 2 × 300 bp paired‐end reads. The Illumina NovaSeq 6000 Reagent Kit and Illunima NovaSeq PE250 were used to sequence the COI fragment (2 × 300 bp paired‐end reads). Libraries were prepared and sequenced by Shanghai Personal Biotechnology Co., Ltd (Shanghai, China).

Sequence assignment, quality control, and initial identifications were carried out using QIIME (v.1.17). The script split_libraries_fastq.py was used to match sequences to individual samples based on Index sequences, while sequences under 10 bp in length and those with *Q*‐values below 20 were excluded (Kuczynski et al., 2011). Sequences were spliced using FLASH (Magoč & Salzberg, 2011), with subsequent discarding of Index sequences. Further quality filtering was performed using the fastq_filter command in USEARCH 11. High‐quality sequences that remained were clustered into operational taxonomic units (OTUs) using UCLUST with a 97% sequence identity threshold (Edgar, 2010). Default parameters were applied to select a representative sequence from each OTU. Species identification was conducted by comparing representative sequences to databases using BLAST (Altschul et al., 1997). COI fragments were annotated using the NCBI database, and ITS2 fragments were annotated through the PLANiTS database (Banchi et al., 2020). Guided by literature on the study site (Long et al., 2010; Zhang & Jiang, 2022), annotated species data within the OTU table underwent individual screening. Taxonomic units corresponding to species with clear distribution records in the study area were retained. Annotations lacking distinct area records were categorized as “Unclassified”. The refined OTU table was then used for subsequent data analyses.

### 2.4 16S rRNA gene Illumina sequencing and processing

To sequence the intestinal bacterial composition, genomic DNA was extracted using the CTAB/SDS method (Caporaso et al., 2012; Doyle & Doyle, 1990). The highly variable V3‐V4 region of the 16S rRNA gene was PCR‐amplified from the extracted DNA using the primers 341F and 806R (Table S3; Caporaso et al., 2012). For the PCR assays, we mixed 15 μL of Phusion Master Mix (2×) (New England Biolabs Inc., China), 3 μL each of the forward and reverse primers (2 μM), 10 μL of DNA template, and 2 μL of ddH_2_O. The PCR amplification parameters were as follows: pre‐denaturation at 98°C for 1 min, denaturation at 98°C for 10 s, annealing at 50°C for 30 s, extension at 72°C for 30 s for 30 cycles, and a final extension at 72°C for 5 min. PCR products were quantified using a Qubit 2.0 Fluorometer (Life Technologies, USA), and their quality for deep sequencing was checked using a Fragment Analyzer (Advanced Analytics Technologies, USA). Libraries were prepared using the Illumina TruSeq DNA Nano Library Prep Kit and were subsequently prepared and sequenced by Health Time Gene Technology Co., Ltd (Shenzhen, China).

The paired‐end sequences of the 16S rRNA genes were assembled using FLASH (v. 1.2.11; Magoč & Salzberg, 2011). Read quality checking and filtering were conducted following the standard operating procedure in QIIME (v. 1.17; Kuczynski et al., 2011). Amplicon sequencing chimeras were eliminated using the UCHIME algorithm (v. 4.2.40; Edgar et al. 2011). We utilized VSEARCH (v. 2.4.4) to clustered the quality‐filtered sequences into OTUs at a sequence similarity threshold of 97% (Rognes et al., 2016). OTUs were assigned and identified to taxa using the SLIVA (v.1.3.2), GreenGene (v.13.8), and Unite (v. 7) reference libraries, and non‐bacterial sequences (e.g., mitochondrial sequences) were removed (Quast et al., 2012).

### 2.5 Statistical analyses

We computed alpha‐diversity indices for gut microbiota using QIIME (v.1.17; Kemp & Aller, 2004). To assess differences in microbial communities across migration stages, we applied Adonis for variance estimation and ANOSIM for testing significant separation between groups (Clarke, 1993). Similarities across migration stages and gut segments were analyzed using Bray–Curtis dissimilarity metric. To identify the most variable groups of gut microorganisms, we performed principal component analysis and ranked bacterial genera based on their median absolute deviation values (Bodawatta et al., 2018). All analyses were conducted using the “FactoMineR” and “factoextra” packages in R v.4.1.3 (Kassambara, 2015; Lê et al., 2008). For functional prediction of the gut microbiota, we employed PICRUSts2 to predict Kyoto Encyclopedia of Genes and Genomes (KEGG) pathways and used the KEGG database to identify metabolic pathway maps of bacteria (Guo et al., 2021). To assess data normality, we performed the Shapiro–Wilk test, and non‐normally distributed data were log‐transformed. The Levene test was used to examine the homogeneity of variances in ANOVA. Statistical significance was set at *p* < .05 to indicate a significant difference.

## 3 RESULTS

### 3.1 Dietary composition and diversity at different migratory stages

In the animal‐based diet identification, we obtained a total of 988,509 raw paired‐end reads and 909,227 high‐quality reads from 12 samples (Table S2). Clustering resulted in a total of 1428 OTUs. For the plant‐based diet, we obtained 913,710 raw paired‐end reads, resulting in 686,738 high‐quality reads and 1465 OTUs (Table S2). The animal‐based dietary reads represented six phyla, 16 classes, 37 orders, and 95 families, while the plant‐based dietary reads represented two phyla, seven classes, 28 orders, and 49 families. Both animal‐based and plant‐based diets displayed seasonal separation (Figure S3).

**FIGURE 2:**
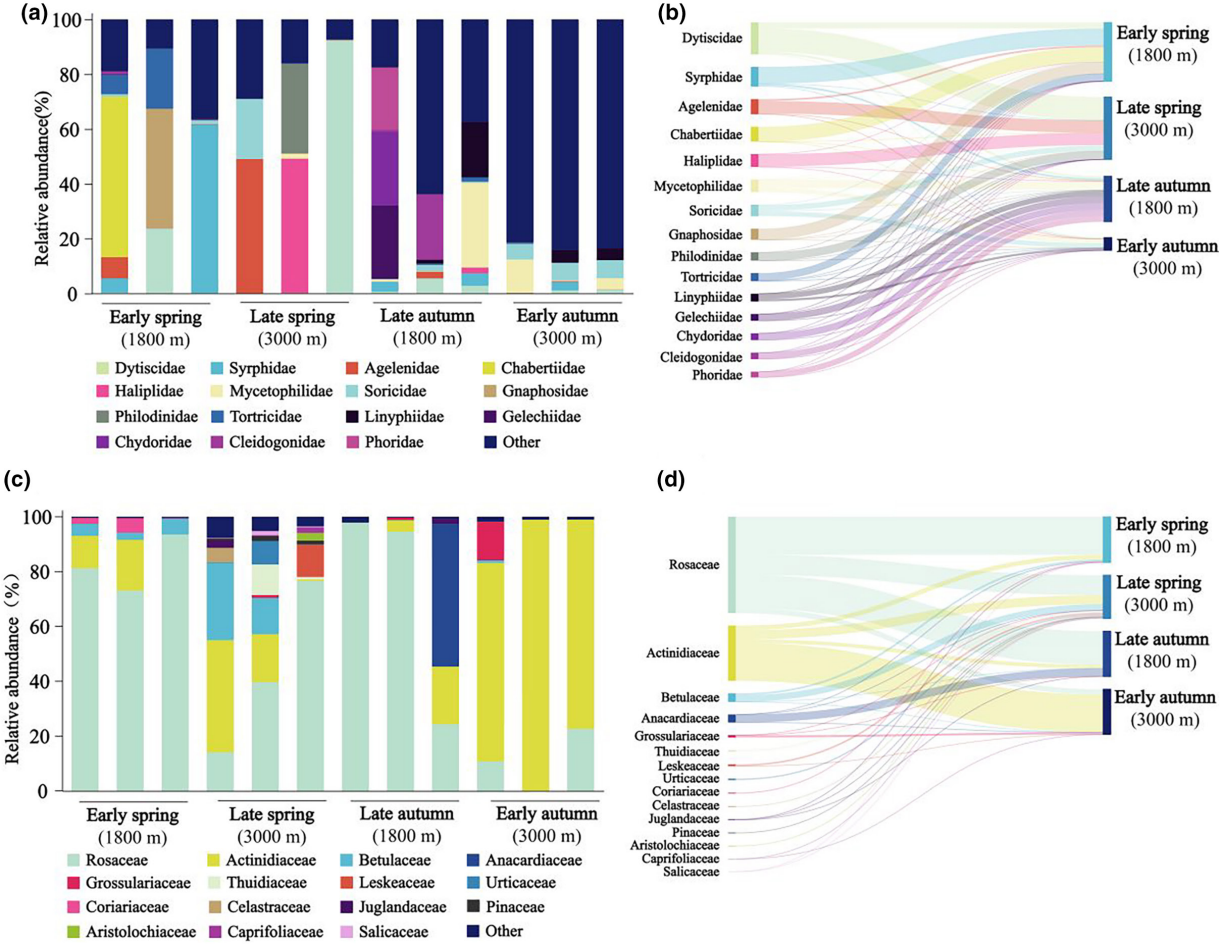
Dietary relative abundance and compositions at the family level in different migration stages. (a) The relative abundance of animal‐based diet. (b) The composition of animal‐based diet. (c) The relative abundance of plant‐based diet. (d) The composition of plant‐based diet. Taxa with relative abundance below TOP15 are grouped together as “others.” Each histogram bar represents the relative abundance for a sample, expressed as the percentage of sequence reads attributable to each family relative to total reads. Sample‐specific information can be found in Table S2.

We observed variations in diversity and relative abundance across different migration periods when comparing animal‐based and plant‐based diets (Figure 2). In the early spring (1800 m), the animal‐based diet primarily consisted of insect families like Syrphidae, spider families such as Lamponidae, and nematode families including Chabertiidae. The plant‐based diet exhibited the highest relative abundance in Rosaceae. In the late spring (3000 m), the main animal‐based diet shifted to include families like water beetles (Dytiscidae and Haliplidae) and spiders (Agelenidae), while the plant‐based diet remained stable. In the early autumn (3000 m), the animal‐based diet showcased insect families such as Mycetophilidae, shrew families like Soricidae, and water flea families such as Chydoridae. The primary plant‐based diet now featured the cashew family, Anacardiaceae. In late autumn (1800 m), the animal‐based diet saw increased relative abundances in water flea families (Chydoridae), millipede families (Cleidogonidae), and insect families (Phoridae). A similar increase was noted in the relative abundance of the plant‐based diet in Rosaceae.

### 3.2 Intestinal microbiota composition and diversity at different migratory stages

**FIGURE 3:**
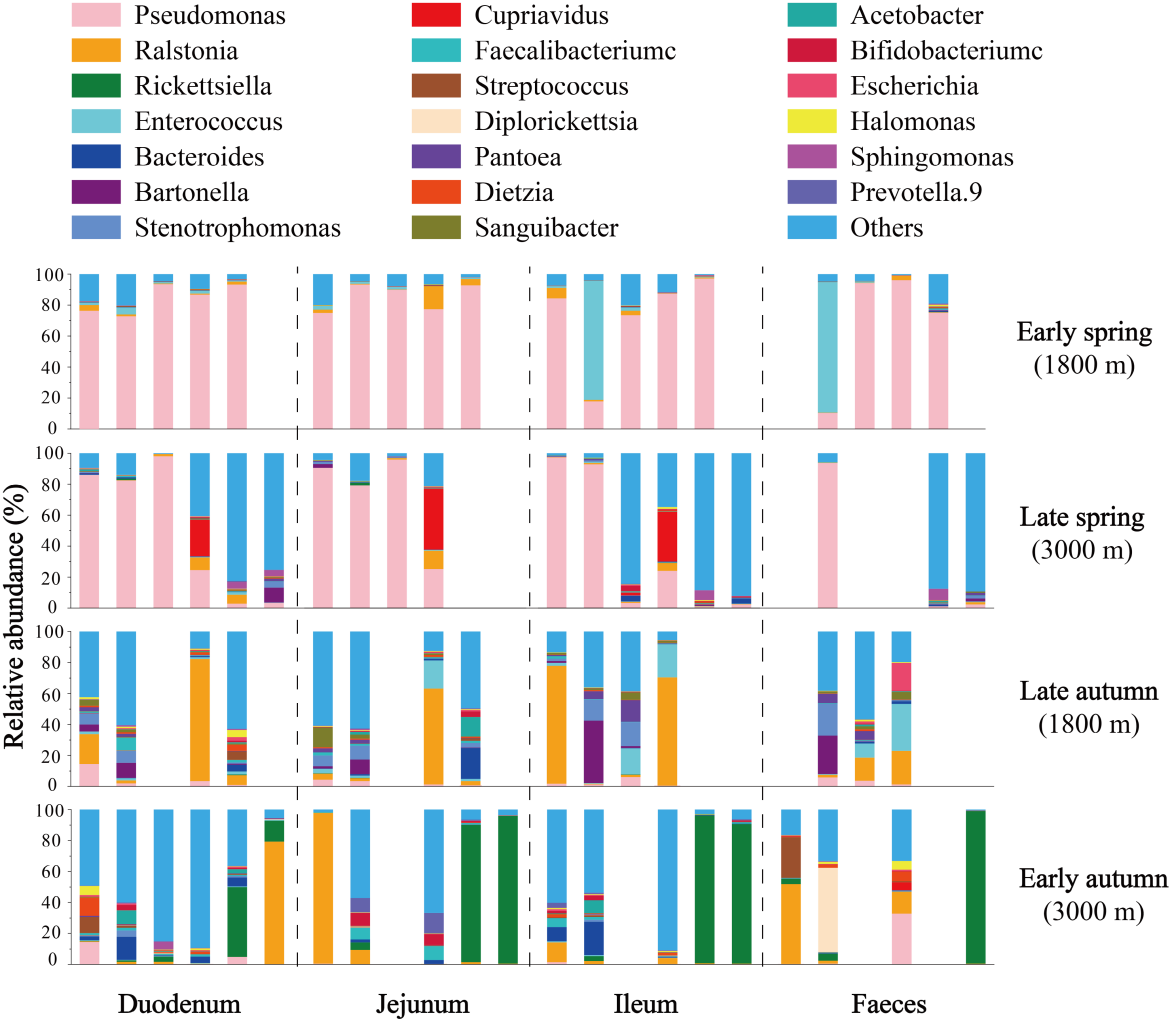
The proportion of major bacterial genus present in the different intestinal segments across different migration stages. Each bar in the histograms representing the relative abundance for an individual, calculated as the percentage of genus‐specific sequence reads out of the total reads. Sample‐specific information can be found in Table S1.

The composition of the intestinal microbiota exhibited significant variations. *Pseudomonas* dominated during spring migration, explaining 78.2% of the variability, while *Ralstonia* dominated during autumn migration, explaining 17.8% of the variability (Figures 3 and S4). Specifically, in early spring, *Pseudomonas* accounted for 72.85%–97.30% of all gut microbial taxa in *T. rufilatus*. During late spring, the proportion of *Cupriavidus* increased (23.55%–39.26%). In early autumn, the dominant taxa were primarily *Ralstonia* (61.69%–78.90%), *Rickettsiella* (88.69%–98.51%), and *Bartonella* (9.50%–40.40%). However, *Enterococcus* (0.37%–30.37%) increased during late autumn (Figure 3). This indicated that gut microbial communities change rapidly among seasons and are more similar within the same season (Figure S5). Additionally, *Lactobacillus*, *Bacteroides*, *Faecalibacterium*, and *Enterococcus* constituted a significant proportion of gut microbial composition during almost all migratory stages (Figure S4).

**FIGURE 4:**
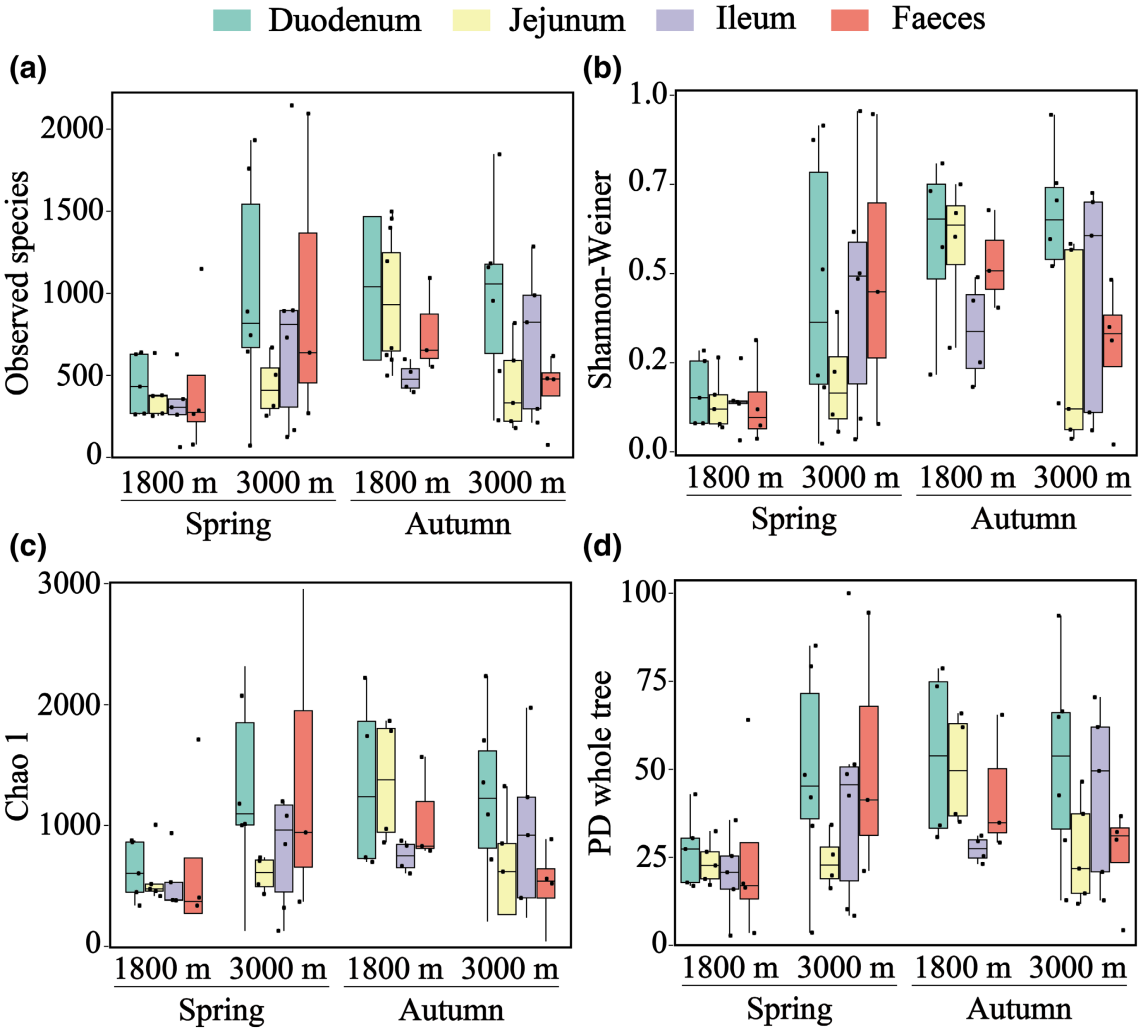
Boxplots of the distribution of alpha‐diversity of microbiota in different migration stages. The boxplots display each diversity index's spread: the median (central line), interquartile range (box edges), and range excluding outliers (whiskers).

Alpha‐diversity was higher in the autumn than in the spring. Across the four migratory stages, the alpha‐index was higher in both of the late stages (Figure 4, Tables S4 and S5). There were no significant differences in the alpha‐diversity of the microbiota among different gut segments. As the migration stage changed, alpha‐diversity varied to different degrees in different gut segments (Figure 4, Tables S4 and S6). However, environmental factors had varying degrees of impact on different intestinal segments of the microbiome. Migratory behavior results in a combination of seasonal and elevational changes. We found that these changes had the greatest effect on the jejunum (*R* ^2^ = 5.226, *p* = .001), while the feces showed the least effect (*R* ^2^ = 1.820, *p* = .018; Table 1).

**TABLE 1:** Adonis and ANOSIM statistical tests on the effects of seasonal and elevation factors on different intestinal microbial segments.

	*N*	Adonis		ANOSIM	
		*R* ^2^	*p*	*R* ^2^	*p*
All samples	22	11.139	.001*	0.481	.001*
Duodenum	21	3.579	.001*	0.391	.002*
Jejunum	18	5.226	.001*	0.572	.001*
Ileum	20	4.342	.001*	0.503	.001*
Faces	14	1.820	.01*	0.385	.004*

*Note*: Sample count (*N*), *R* ^2^ values, and *p* values are shown. An asterisk (*) denotes *p* < .05, indicating statistical significance.

### 3.3 Diet at different migratory stages correlates with gut microbiota function

**FIGURE 5:**
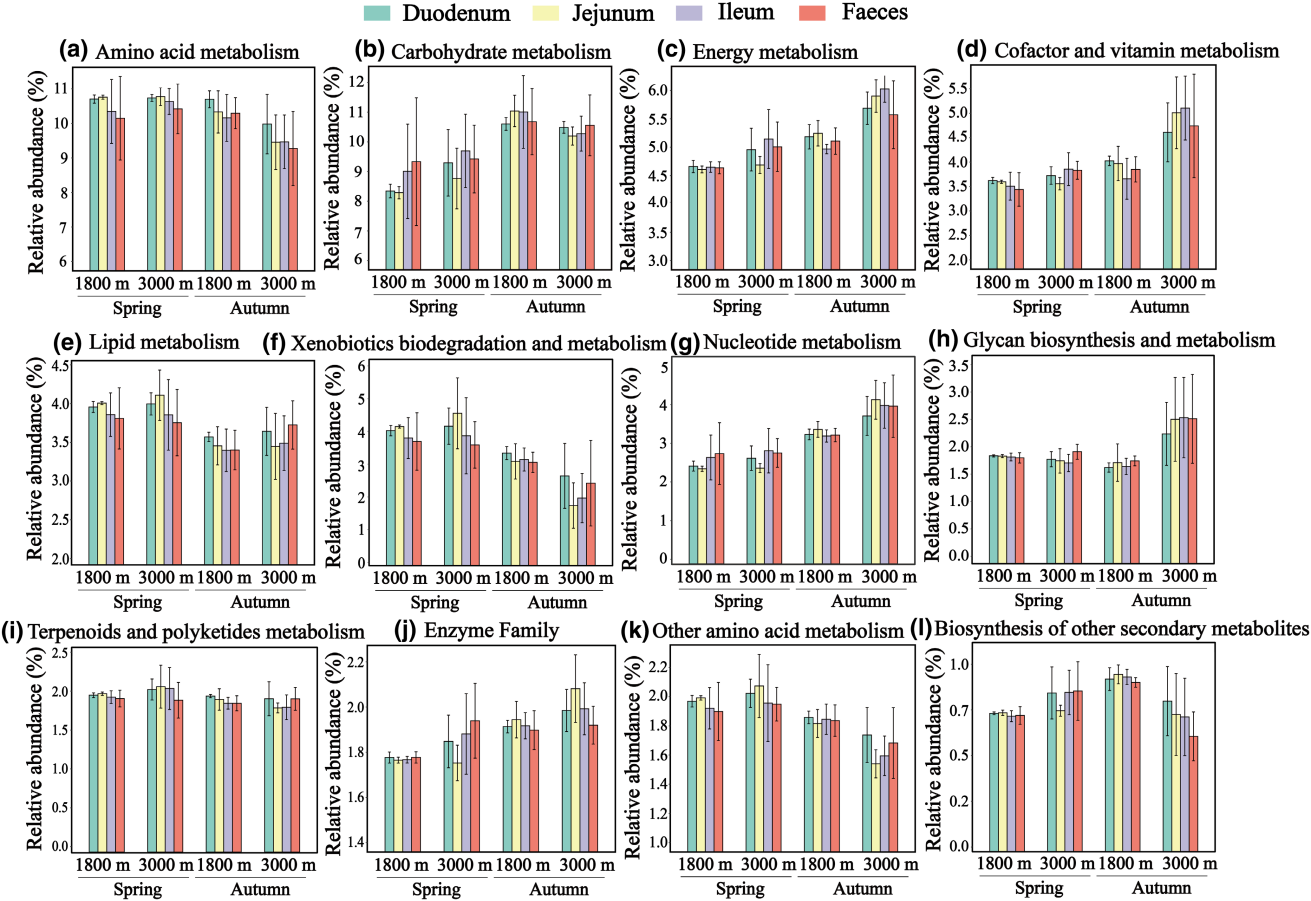
Functional abundance of the KEGG level 2 metabolic pathway in the intestinal of Himalayan Bluetail. The whiskers on the bar graphs represent the standard deviation from the mean.

The dominant taxon across different migration stages, as indicated by variations in the relative abundance of gut microorganisms, is *Pseudomonas* (Figures 3 and S4). Analysis based on the KEGG reveals that *Pseudomonas* plays a significant role in enriching enzymes related to amino acid and fatty acid biosynthesis pathways (Figure S6). Furthermore, we observed that the relative abundance of 11 metabolic pathways significantly differed across migration stages. Five metabolic pathways, including amino acid metabolism, lipid metabolism, xenobiotics biodegradation and metabolism, glycan biosynthesis and metabolism, and other amino acid metabolism, were more abundant in spring. Conversely, the other six pathways were more abundant in autumn. During early autumn (3000 m), out of these six pathways, four pathways (energy, cofactors, nucleotide metabolism, and enzyme families) exhibited highly abundance (Figure 5b–d,g,i–l, Tables S7 and S8). The enzyme family showed significant differences (*p* < .05) among intestinal segments, displaying an increasing trend during digestion in both seasons, except for the lowest spring abundance in the jejunum (Figure 5j).

### 3.4 Correlation of diet–gut microbes in different intestinal segments

**FIGURE 6:**
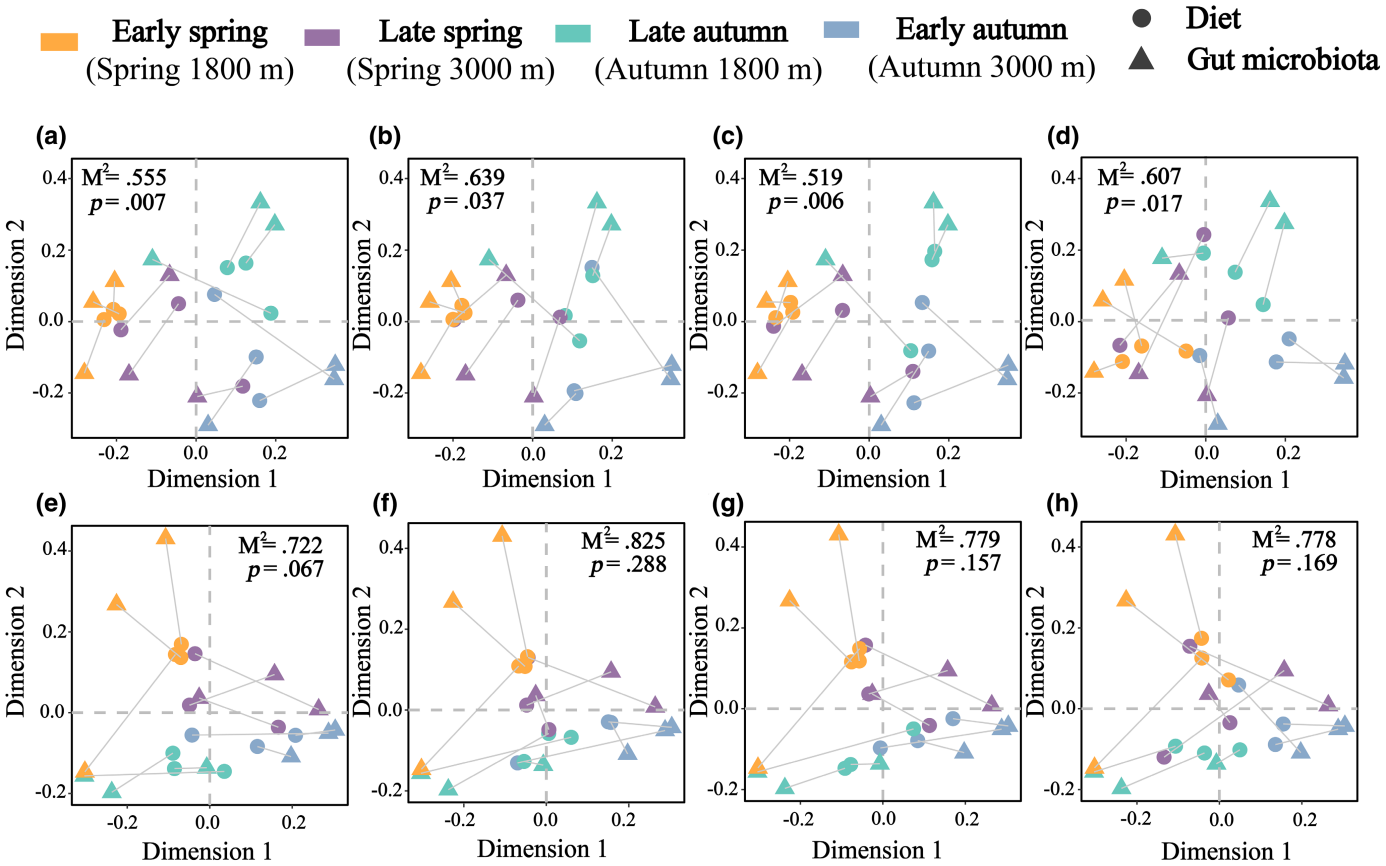
Procrustes analysis of the correlation between diet and gut microbial communities. (a–d) are animal‐based diet in the duodenum, jejunum, ileum, and feces. (e‐h) are plant‐based diet in the duodenum, jejunum, ileum, and feces.

**FIGURE 7:**
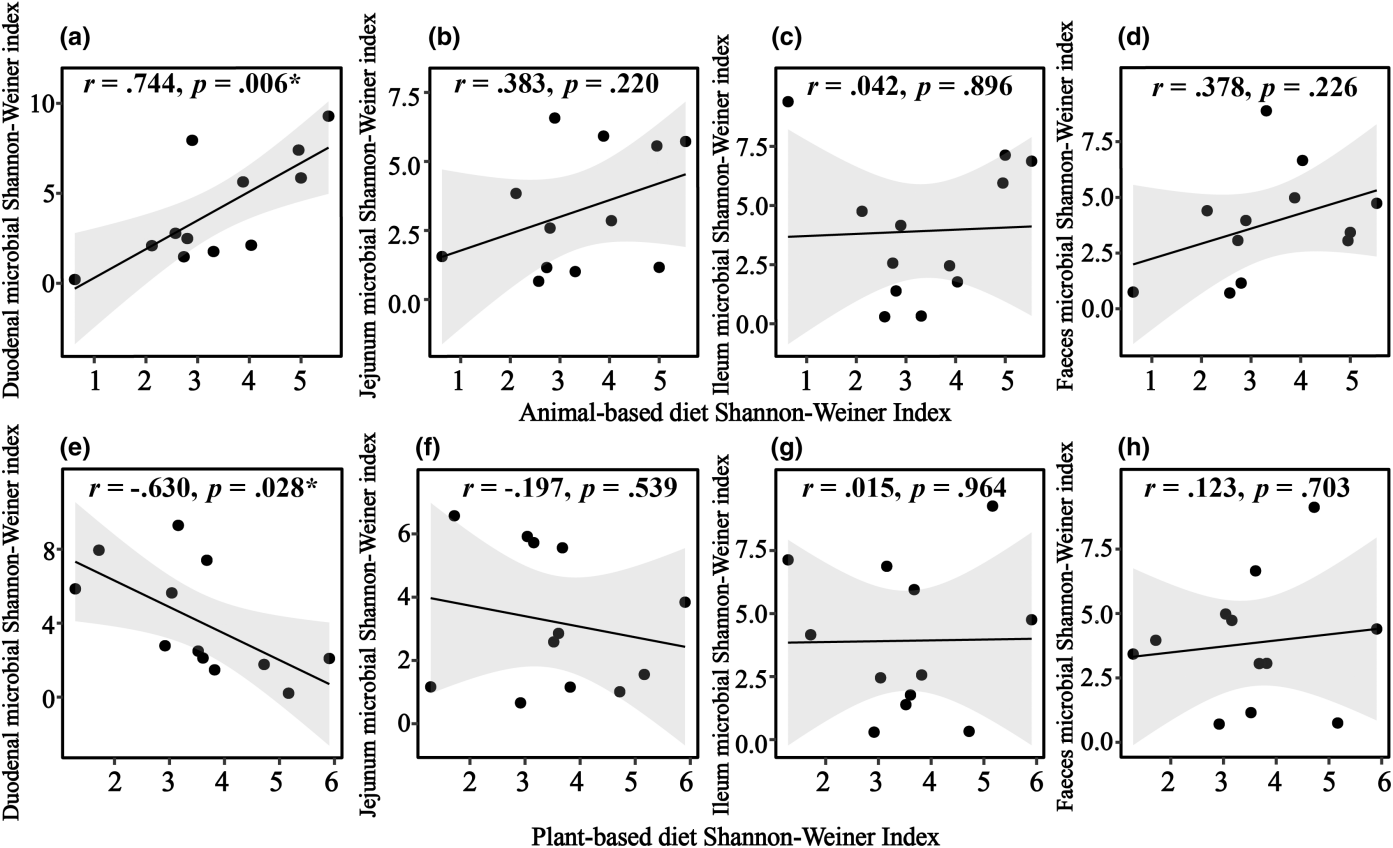
Linear regression analysis of intestinal microbial diversity and plant and animal food diversity.

We observed a significant correlation between the animal‐based diet and microbial composition in all intestinal segments, while the plant‐based diet showed a weaker correlation in each segment (Figure 6). The duodenum, jejunum, and feces exhibited a positive correlation between microbial diversity and the animal‐based diet (Figure 7a,b,d), but showed a negative correlation with the plant‐based diet diversity (Figure 7e,f,h). However, the correlation between ileal microbial diversity and both the animal‐based and plant‐based diets was not found to be significant (Figure 7c,h).

## 4 DISCUSSION

In this study, we conducted the first investigation into the seasonal dynamics of diet–gut interactions within *Tarsiger rufilatus*, a migratory songbird known for its elevational movements. This species experiences year‐round elevational shifts and endures drastic environmental variations such as hypoxia, hypothermia, and limited food availability. Surviving under these conditions necessitates diverse and responsive gut microbes as a potential adaptation.

### 4.1 Migration behavior effects on rapid changes in gut microbial composition and function

Our findings revealed that the elevational migration pattern leads to rapid alterations in both gut microbial composition and function, occurring within a single month. Similar rapid microbial variations have been observed in various animal taxa, within as short as 1–11 days (Buglione et al., 2022; David et al., 2014; Hao et al., 2017; Lewis et al., 2017). Seasonal movements trigger shifts in vegetation and food availability, thereby influencing the composition of gut microbes. This is consistent with findings in long‐distance migrating birds (Guo et al., 2021; Maurice et al., 2015; Risely et al., 2018). Moreover, avian foraging in the wild leads to the intake of environmental microorganisms that can affect gut microbial composition (Risely et al., 2017). Alterations in gut microbial composition result from multifaceted interactions, contributing to host health across diverse environments. Food‐related microbes remain pivotal in shaping the potential impact of the gut microbiome.

### 4.2 Seasonal diet shifts shape gut microbiota interactions during migration

We observed interactions between the diet and gut microbiota at various migration stages. As an insectivorous bird, *T. rufilatus* maintains a consistent core gut microbial composition similar to that of finches (Figure 3; Bodawatta et al., 2018; Grond et al., 2018). Besides these core microbiotas, the composition of other gut microbiota varied with changes in seasonal diet composition. In the early spring migration, characterized by low temperatures and limited food availability, there is a noticeable decrease in gut microbial alpha‐diversity (Figures 4 and S4; Table S5). During this period, a uniform gut microbial composition was dominated by *Pseudomonas*. This bacterium produces lipases that aid in energy extraction from dietary fats, which is particularly beneficial in cold environments (Rosenau & Jaeger, 2000). Moreover, the alignment between digestive demands and gut microbial metabolic function was pronounced in both seasons. Specific taxa associated with animal‐based foods were relatively more prevalent in the spring, while taxa associated with plant‐based foods were more prevalent in the autumn (Figure 2). Consequently, amino acid metabolism, xenobiotics biodegradation and metabolism were prominent in spring (Figure 5a,f), whereas carbohydrate metabolism, cofactor and vitamin metabolic pathways were abundant in autumn (Figure 5b,d). These results suggest that the functions of microbial communities, as well as their interactions with hosts and the environment, contribute to their ability to cope with environmental shifts. In our study, we found high abundances of Lepidoptera and Diptera in *T. rufilatus*' animal‐based diet during different migratory stages, consistent with its known preferences (del Hoyo et al., 2023). However, potential overestimation of these taxa due to bias in the ZBJ marker was noted (Forsman et al., 2022). Despite this, our analysis still showed a significant correlation between animal‐based foods and gut microbiome composition. Future research should explore diverse molecular markers and metagenomics techniques to address these biases. This will enhance our understanding of diet‐microbiome interactions and their implications for migratory species' ecological and physiological adaptations, shedding light on how dietary shifts and environmental challenges shape microbial communities.

Notably, the unexpected presence of shrews and nematodes in the diet of *T. rufilatus* contradicts its known feeding habits (del Hoyo et al., 2023). This discrepancy may arise from accidental predation or parasitic infections. Birds may inadvertently ingest these organisms while foraging for carrion insects or during fecal ingestion. Additionally, the identification of Strongylida nematodes in stomach contents during field surveys reinforces the influence of parasitic infections on the diet composition. Ascaris infections have been documented in other domestic animals and birds (Thamsborg et al., 2017), providing further evidence for this interpretation. The detection of algae and mosses in the diet may suggest secondary ingestion, where *T. rufilatus* consumes these components indirectly through herbivorous insects (e.g., Lepidoptera larvae) that may carry plant residues, potentially impacting the results of food analysis.

### 4.3 Potential adaptive changes in gut microbiota during high‐altitude migration

Our findings revealed that the gut microbial composition of *T. rufilatus* exhibits convergent responses with other high‐altitude breeding species. Survival at high altitudes necessitates gut microbes to adapt their metabolic functions to cope with cold and oxygen‐deprived environments (Sun et al., 2023). We observed an increased prevalence of amino acid metabolism within the gut microbiota during migration to colder, resource‐scarce areas, enhancing energy extraction and nutrient utilization (Montoya‐Ciriaco et al., 2020; Sears, 2005), consistent with synergistic diet‐microbiota‐metabolism traits observed in high‐altitude *Passer montanus* (Sun et al., 2023). Furthermore, across various migratory stages, we consistently identified dominant bacterial groups such as *Lactobacillus*, *Bacteroides*, *Faecalibacterium*, and *Enterococcus* in *T. rufilatus* (Figure S4). This prevalent phenomenon in high‐altitude breeding birds may play pivotal roles in regulating nutrient metabolism, enhancing nutrient absorption, and promoting immune regulation (Bo et al., 2022; Zocco et al., 2007), suggesting that avian gut microbes respond similarly in comparable high‐altitude habitats. However, the gut microbiota of wild birds, especially migrators, varies with diet, life history, and season due to intricate interactions during high‐altitude acclimatization (Liu et al., 2021). Therefore, additional physiological and metabolic methods are necessary to comprehensively understand the avian diet–gut microbiota relationship and its role in mediating host metabolic traits in high‐altitude environments. Additionally, understanding variations across different intestinal segments is also crucial for studying the effects of the environment on gut microbes.

### 4.4 Seasonal variations in gut microbial diversity across different intestinal segments

Avian gut microbe research has predominantly relied on fecal samples, potentially limiting the depth of insights. Our findings highlighted variations in microbial abundance across different intestinal segments (Figures 3 and 4), likely due to unique anatomical structures and physiological roles of each segment (Kohl et al., 2018). The duodenum, following the stomach, continues digestion, whereas the jejunum and ileum are engaged in digestion and nutrient absorption (Yen, 2000). The higher microbial abundance observed in the duodenum during early spring could be supporting efficient food digestion and nutrient utilization. Interestingly, our results indicate that the jejunal microflora, located in the middle part of the small intestine, displays heightened susceptibility to external factors (Table 1, Figure S2). This susceptibility can be attributed to the extended retention of food in the jejunum compared to the duodenum due to its greater length (Tancharoenrat et al., 2014). Nevertheless, our findings did not reveal significant differences in metabolic capacities among the various intestinal segments (Figure 5, Table S8). This lack of differentiation may result from functional overlap within the small intestine (Zhang et al., 2020). These results indicate that the microbial communities could be adapting metabolically across migration stages, responding to environmental changes while retaining their core metabolic abilities. Consequently, a comprehensive analysis of microorganism distribution across different intestinal segments and their interactions with hosts is imperative for understanding the plasticity and variability in gut microbial composition.

## AUTHOR CONTRIBUTIONS

**Shangmingyu Zhang:** Conceptualization (lead); data curation (supporting); formal analysis (supporting); investigation (lead); methodology (equal); project administration (equal); visualization (supporting); writing – original draft (lead); writing – review and editing (lead). **Chuang Zhou:** Data curation (supporting); formal analysis (supporting); methodology (supporting); writing – review and editing (supporting). **Zhehan Dong:** Conceptualization (equal); data curation (supporting); formal analysis (supporting); investigation (equal); methodology (equal); project administration (supporting); resources (equal); visualization (equal). **Kaize Feng:** Data curation (supporting); formal analysis (supporting); investigation (supporting); resources (supporting). **Kexin Peng:** Formal analysis (supporting); resources (supporting). **Zhengyang Wang:** Conceptualization (supporting); writing – review and editing (supporting). **Yong Jiang:** Data curation (supporting); investigation (supporting). **Linyu Jin:** Funding acquisition (supporting). **Ping Zhang:** Funding acquisition (supporting). **Yongjie Wu:** Conceptualization (equal); funding acquisition (lead); resources (lead); supervision (lead); writing – original draft (supporting); writing – review and editing (supporting).

## FUNDING INFORMATION

This study was supported by The National Natural Science Foundation of China (No. 32270454), The Second Tibetan Plateau Scientific Expedition and Research Program (No. 2019QZKK0501), the Fundamental Research Funds for the Central Universities and Chengdu Tianfu International Airport Branch of Sichuan Airport Group Limited Company.

## CONFLICT OF INTEREST STATEMENT

The authors declare no competing interests.

## Supporting information


Data S1.

## Data Availability

The data that support the findings of this study are openly available in Dryad at https://doi.org/10.5061/dryad.1jwstqk1n (Zhang et al. 2024).
