# Supplementary material for: The diet–intestinal microbiota dynamics and adaptation in an elevational migration bird, the Himalayan bluetail (Tarsiger rufilatus)
Source: Ecol Evol. 2024 Jun 29;14(7):e11617. doi: 10.1002/ece3.11617 (PMC11214064; doi:10.1002/ece3.11617)
Supplement: Supplementary file 1 — Data S1. [file ECE3-14-e11617-s001.zip › Supplemental Materials.docx]

# SUPPLEMENTAL FIGURES

**Supplemental figure S1:**

**
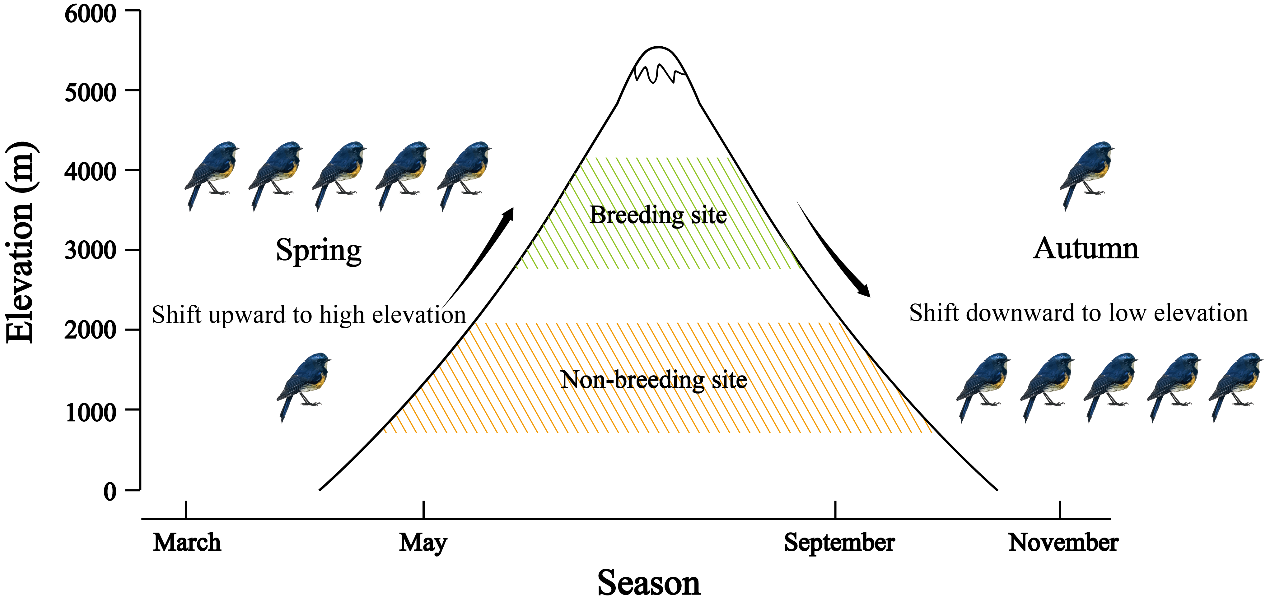
**

**Figure S1.** Elevational migration of the Himalayan Bluetail (*Tarsiger rufilatus*)

**Supplemental figure S2:**

**
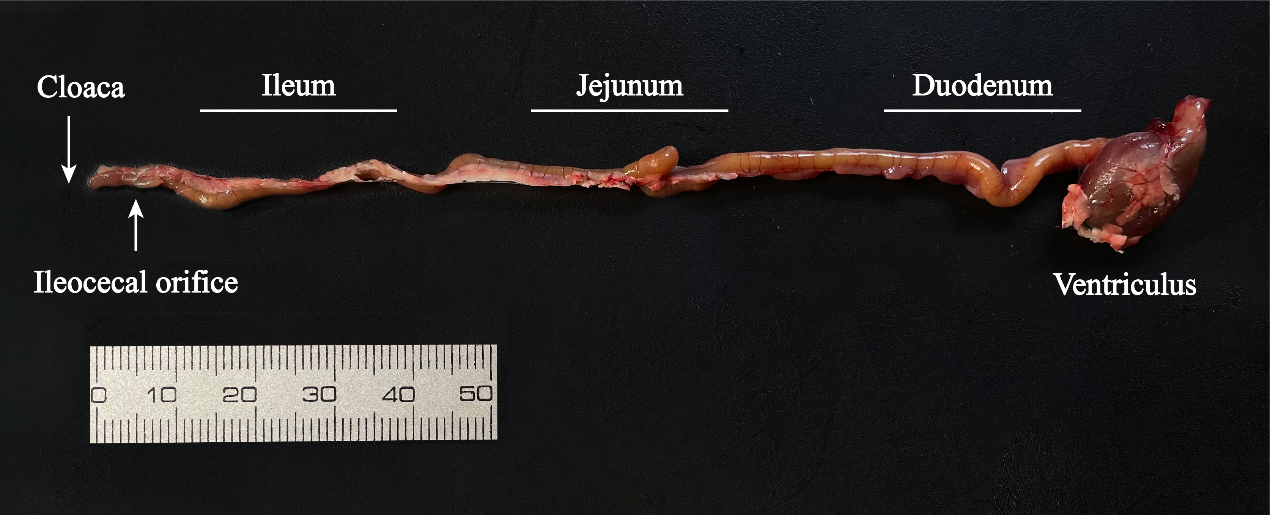
**

**Figure S2.** Three intestinal segments of the Himalaya Bluetail used for microbiota analysis.

**Supplemental figure S3:**


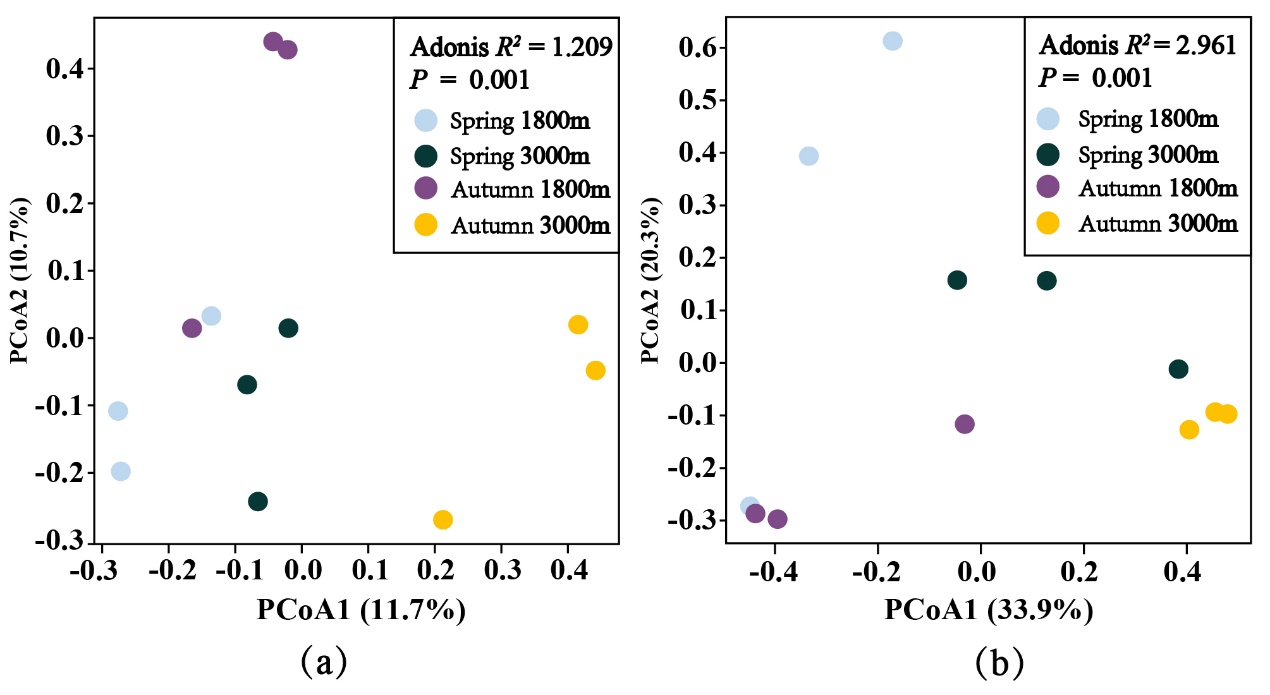


**Figure S3.** Diets of Himalayan Bluetail clustered using PCoA of the Bray-Curtis distance matrix. The percentage of variation explained by the plotted principal coordinates is indicated on the axes. Each point corresponds to a sample colored by (A) animal-based diets, (B) plant-based diets.

**Supplemental figure S4:**


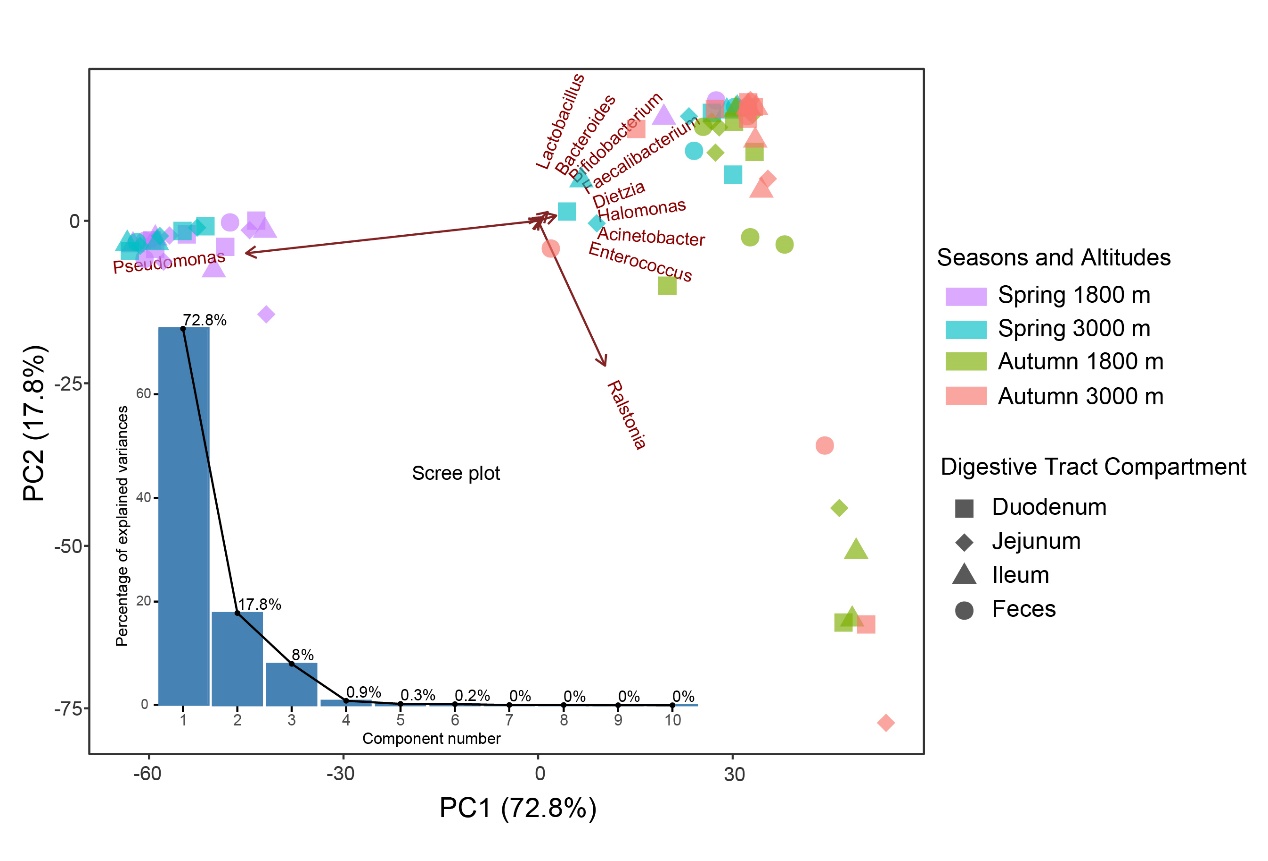


**Figure S4** Principal component analysis (PCA) of gut microbiota in different intestinal segments across migration stages. The scree plot represents the variation in the data captured by the first 10 principal components.

**Supplemental figure S5:**


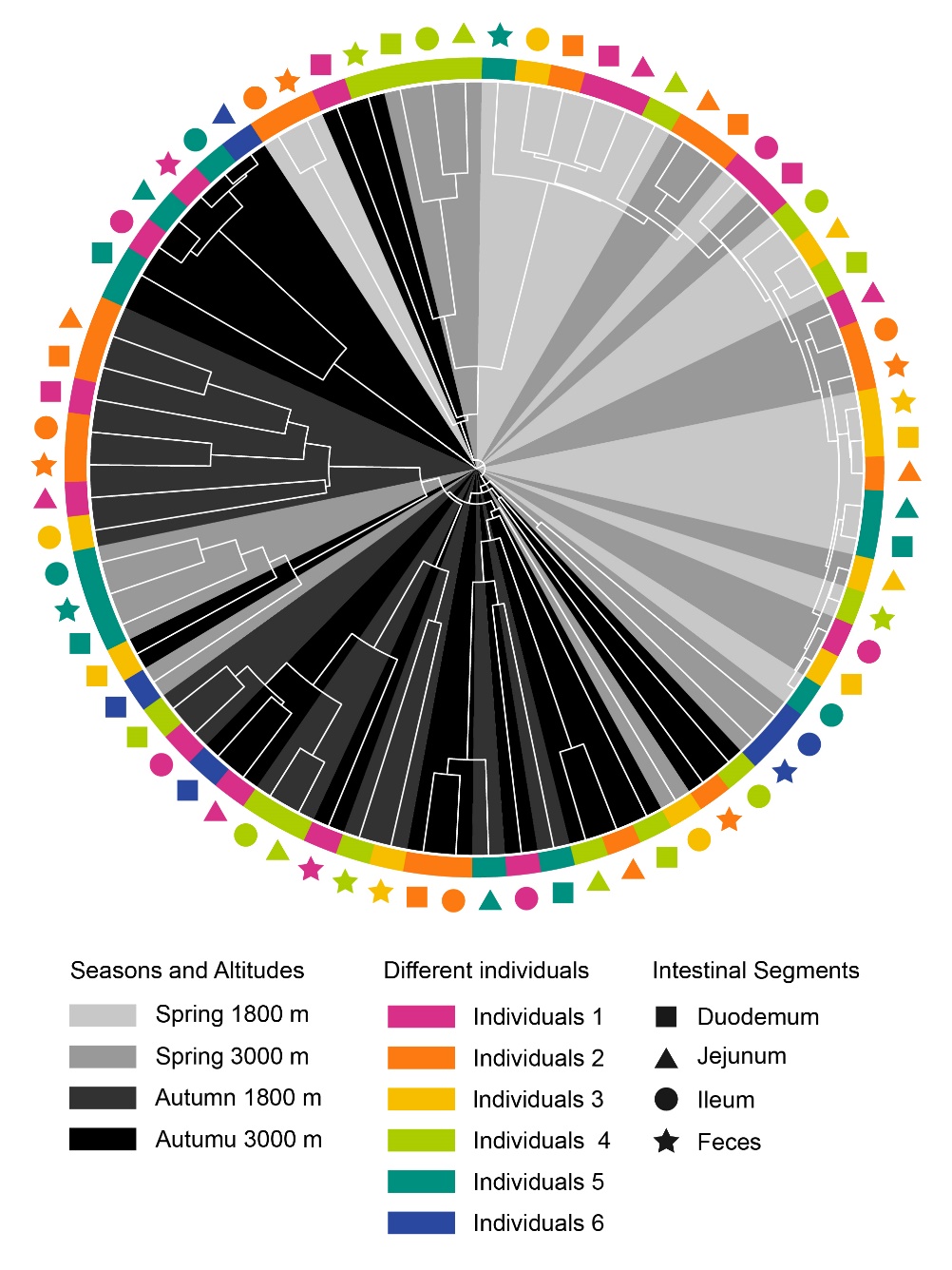


**Figure S5** Bray-Curtis dissimilarity of gut microbiota between different intestinal segments across migration stages.

**Supplemental figure S6:**


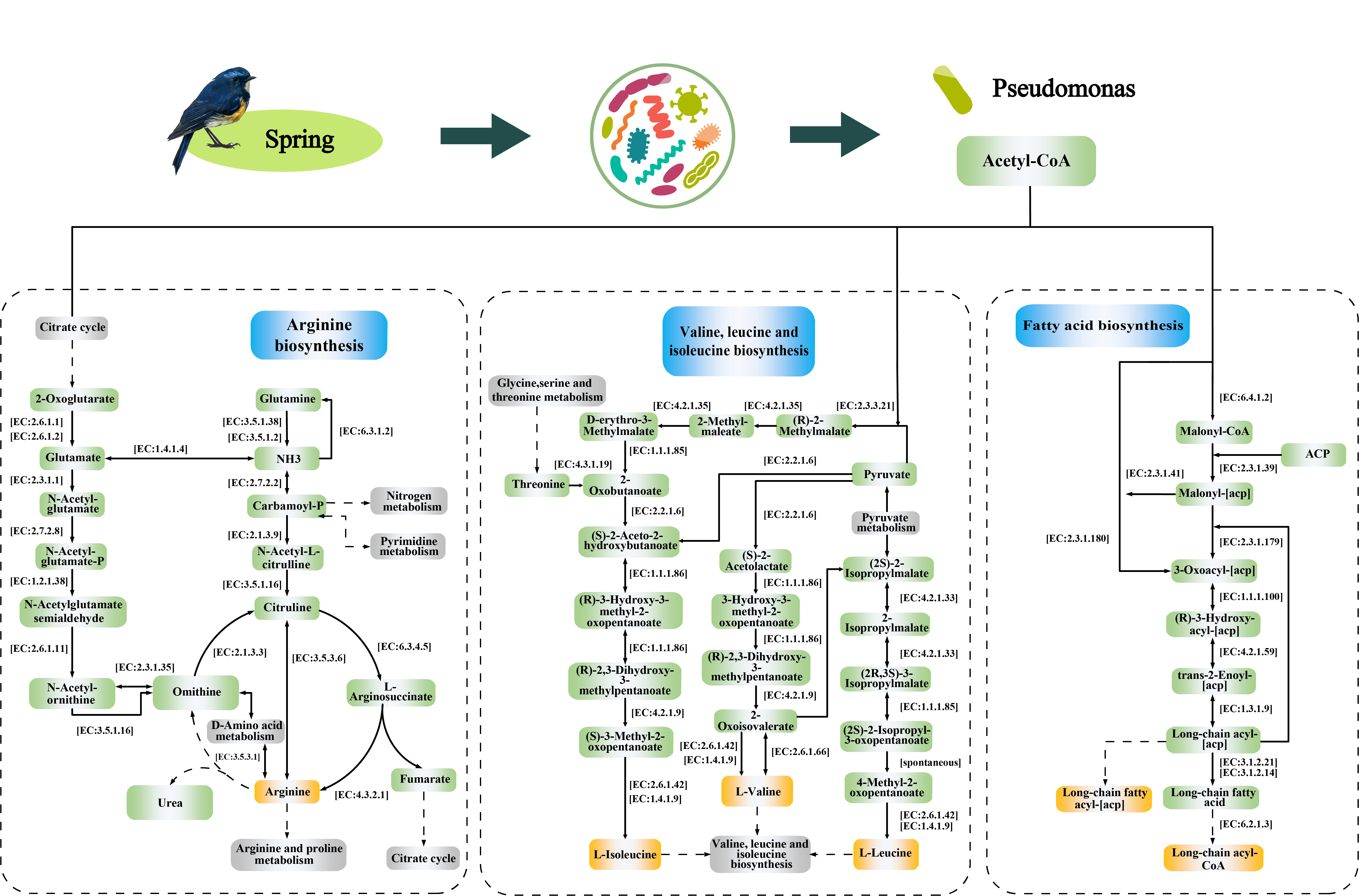


**Figure S6** Metabolic pathway associated with Pseudomonas on host metabolism. The dotted boxes from left to right are arginine biosynthesis, valine, leucine and isoleucine biosynthesis, and fatty acid biosynthesis.

**Supplemental figure S7:**


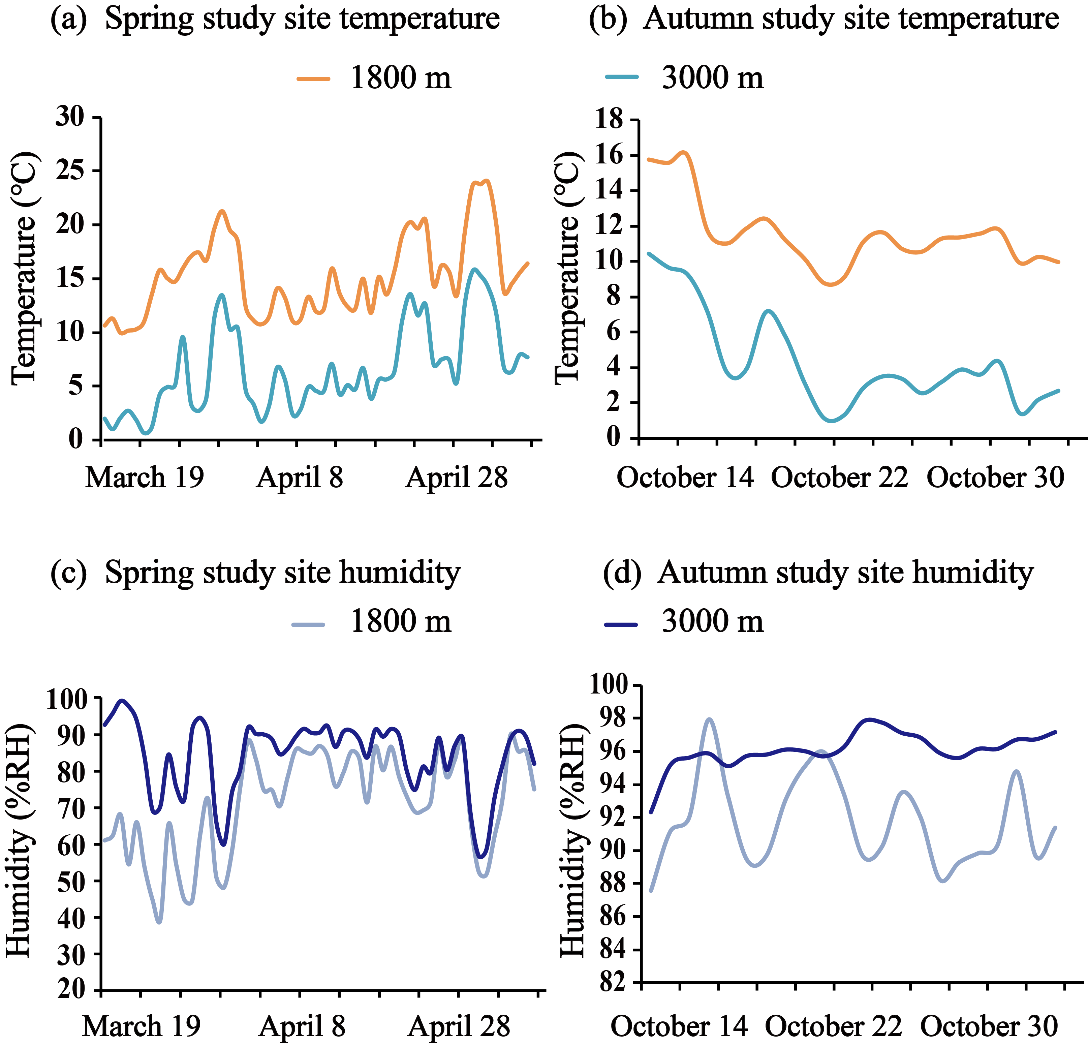


**Figure S7** Temperature and relative humidity of the sampling site
