# Supplementary material for: The diet–intestinal microbiota dynamics and adaptation in an elevational migration bird, the Himalayan bluetail (Tarsiger rufilatus)
Source: Ecol Evol. 2024 Jun 29;14(7):e11617. doi: 10.1002/ece3.11617 (PMC11214064; doi:10.1002/ece3.11617)
Supplement: Supplementary file 1 — Data S1. [file ECE3-14-e11617-s001.zip › Supplemental Tables.docx]

# SUPPLEMENTAL TABLES

**Table S1** Himalayan Bluetail intestinal microbiota samples information. “S”, Spring; “A”, Autumn; “L”, 1800 m; “H”, 3000 m. “*” indicates samples of intestinal contents that have passed quality control for subsequent analysis.

| **No.** | **Sample ID** | **Sex** | **Elevation** | **Season** | **Date** | **Intestine segment** | | | |
| --- | --- | --- | --- | --- | --- | --- | --- | --- | --- |
|  |  |  |  |  |  | **Duodenum** | **Jejunum** | **Ileum** | **Faeces** |
| 1 | SL1 | Male | 1800 m | Spring | 2021.3.14 | * | * | * |  |
| 2 | SL2 | Male | 1800 m | Spring | 2021.3.21 | * | * | * | * |
| 3 | SL3 | Male | 1800 m | Spring | 2021.4.8 | * | * | * | * |
| 4 | SL4 | Male | 1800 m | Spring | 2021.4.13 | * | * | * | * |
| 5 | SL5 | Male | 1800 m | Spring | 2021.4.16 | * | * | * | * |
| 6 | SH1 | Male | 3000 m | Spring | 2021.4.12 | * | * | * |  |
| 7 | SH2 | Female | 3000 m | Spring | 2021.4.12 | * | * | * | * |
| 8 | SH3 | Female | 3000 m | Spring | 2021.4.13 | * | * | * |  |
| 9 | SH4 | Male | 3000 m | Spring | 2021.4.19 | * | * | * |  |
| 10 | SH5 | Male | 3000 m | Spring | 2021.4.28 | * |  | * | * |
| 11 | SH6 | Male | 3000 m | Spring | 2021.5.7 | * |  | * | * |
| 12 | AL1 | Male | 1800 m | Autumn | 2021.10.30 | * | * | * |  |
| 13 | AL2 | Male | 1800 m | Autumn | 2021.11.1 | * | * | * | * |
| 14 | AL3 | Male | 1800 m | Autumn | 2021.11.1 |  |  | * | * |
| 15 | AL4 | Male | 1800 m | Autumn | 2021.11.2 | * | * | * | * |
| 16 | AL5 | Male | 1800 m | Autumn | 2021.11.2 | * | * |  |  |
| 17 | AH1 | Female | 3000 m | Autumn | 2021.10.12 | * | * | * | * |
| 18 | AH2 | Female | 3000 m | Autumn | 2021.10.13 | * | * | * | * |
| 19 | AH3 | Male | 3000 m | Autumn | 2021.10.14 | * |  |  |  |
| 20 | AH4 | Female | 3000 m | Autumn | 2021.10.15 | * | * | * | * |
| 21 | AH5 | Male | 3000 m | Autumn | 2021.10.15 | * | * | * |  |
| 22 | AH6 | Male | 3000 m | Autumn | 2021.10.19 | * | * | * | * |

**Table S2**. List of the effective sequence number and OTUs number for each sample. “S”, Spring; “A”, Autumn; “L”, 1800 m; “H”, 3000 m.

| No. | Sample ID | Animal-based diet | | Plant-based diet | |
| --- | --- | --- | --- | --- | --- |
|  |  | Raw PE  sequences | High-quality  sequences | Raw PE  sequences | High-quality sequences |
| 1 | AH1 | 69516 | 61521 | 20034 | 17610 |
| 2 | AH2 | 91079 | 80666 | 83477 | 55038 |
| 3 | AH3 | 96029 | 83202 | 72591 | 60670 |
| 4 | AL1 | 67521 | 63315 | 70077 | 44227 |
| 5 | AL2 | 107223 | 99047 | 75302 | 46374 |
| 6 | AL3 | 125808 | 114835 | 94960 | 64192 |
| 7 | SH1 | 82324 | 77819 | 108844 | 99253 |
| 8 | SH2 | 66702 | 63802 | 69752 | 57711 |
| 9 | SH3 | 61762 | 59514 | 87770 | 63168 |
| 10 | SL1 | 83953 | 76711 | 78184 | 54175 |
| 11 | SL2 | 63862 | 61425 | 92756 | 83380 |
| 12 | SL3 | 72730 | 67370 | 59963 | 40940 |
| Total | | 988509 | 909227 | 913710 | 686738 |

**Table S3** Primers utilized in this study

| **Primer** | **Sequence** | **Refence** |
| --- | --- | --- |
| ZBJ-ArtF1c | AGATATTGGAACWTTATATTTTATTTTTGG | Zeale et al., 2011 |
| ZBJ-ArtR2c | WACTAATCAATTWCCAAATCCTCC |  |
| ITS2-F | ATGCGATACTTGGTGTGAAT | Tripathi et al., 2013 |
| ITS2-R | GACGCTTCTCCAGACTACAAT |  |
| 341F | CCTAYGGGRBGCASCAG | Lun et al., 2019 |
| 806R | GGACTACHVGGGTWTCTAAT |  |

**Table S4**. Three-factor ANOVA of α-diversity index of intestinal microbiota

| **α-diversity index** | **Statistical results** | | |
| --- | --- | --- | --- |
|  | **Factors** | **F** | **p** |
| Observed species | Season  Elevation  Intestinal segments  Season $\times$Elevation  Season$\times$Intestinal segments  Elevation$\times$Intestinal segments  Season$\times\mathrm{Elevation}\times$Intestinal segments | 2.727  0.136  1.257  6.644  0.217  1.033  0.443 | 0.104  0.714  0.298  **0.013**  0.884  0.385  0.723 |
| Shannon | Season  Elevation  Intestinal segments  Season $\times$Elevation  Season$\times$Intestinal segments  Elevation$\times$Intestinal segments  Season$\times\mathrm{Elevation}\times$Intestinal segments | 8.722  0.053  0.828  7.701  0.229  0.812  0.546 | **0.005**  0.820  0.484  **0.007**  0.876  0.492  0.653 |
| Chao1 | Season  Elevation  Intestinal segments  Season $\times$Elevation  Season$\times$Intestinal segments  Elevation$\times$Intestinal segments  Season$\times\mathrm{Elevation}\times$Intestinal segments | 2.182  0.042  1.085  8.296  0.297  0.936  0.449 | 0.145  0.838  0.363  **0.006**  0.827  0.430  0.719 |
| PD whole tree | Season  Elevation  Intestinal segments  Season $\times$Elevation  Season$\times$Intestinal segments  Elevation$\times$Intestinal segments  Season$\times\mathrm{Elevation}\times$Intestinal segments | 2.895  0.370  1.102  5.703  0.264  1.199  0.545 | 0.094  0.545  0.356  **0.020**  0.851  0.318  0.654 |

Table S5. Gut microbial alpha-diversity means at different migration stages. “L”, 1800 m; “H”, 3000 m.

| **Migration stage** | **Observed_species** | **Shannon** | **Chao1** | **PD_whole_tree** |
| --- | --- | --- | --- | --- |
| **Spring** | 631.1052632 | 2.69 | 853.015068 | 37.61283947 |
| **Autumn** | 741.4285714 | 4.476171429 | 994.839669 | 44.44830286 |
| **Spring-L** | 420.9473684 | 1.430426316 | 626.527621 | 28.94384211 |
| **Spring-H** | 841.2631579 | 3.949573684 | 1079.50252 | 46.28183684 |
| **Autumn-L** | 825 | 4.9586 | 1130.94777 | 47.4837 |
| **Autumn-H** | 678.75 | 4.11435 | 892.75859 | 42.171755 |

Table S6. Intestinal segments microbial alpha-diversity means at different migration stages. “L”, 1800 m; “H”, 3000 m.

| **Alpha-diversity** | **Intestinal segments** | **Spring** | | **Autumn** | |
| --- | --- | --- | --- | --- | --- |
|  |  | **Spring-L** | **Spring-H** | **Autumn-L** | **Autumn-H** |
| **Observed_species** | Duodenum | 1.447238 | 3.866394 | 4.843257 | 4.731482 |
|  | Jejunum | 1.333081 | 2.0843 | 4.910286 | 4.422182 |
|  | Ileal | 1.189913 | 4.24165 | 4.43441 | 4.465265 |
|  | Feces | 1.221108 | 5.135615 | 3.877657 | 4.056665 |
| **Shannon** | Duodenum | 599.0279 | 1080.222 | 1090.027 | 973.1748 |
|  | Jejunum | 576.7947 | 623.1637 | 1156.207 | 967.8902 |
|  | Ileal | 521.7197 | 1112.657 | 1097.197 | 978.119 |
|  | Feces | 580.9417 | 1257.982 | 885.76 | 932.281 |
| **Chao1** | Duodenum | 403.875 | 848.6471 | 797.8571 | 753.8824 |
|  | Jejunum | 379.9375 | 438.0909 | 838.2857 | 742.5294 |
|  | Ileal | 344.4375 | 890.125 | 777.8 | 749.3529 |
|  | Feces | 389.6154 | 1029.769 | 618 | 705.8235 |
| **PD_whole_tree** | Duodenum | 28.13404 | 45.85292 | 46.26372 | 45.87536 |
|  | Jejunum | 26.72567 | 29.02733 | 48.1292 | 45.43291 |
|  | Ileal | 24.98926 | 47.69825 | 45.37013 | 45.93256 |
|  | Feces | 27.79382 | 54.52788 | 39.13974 | 43.43952 |

Table S7. PICRUSTs2 predicts the functional relative abundance of KEGG Level2 metabolic pathways at different migration stages. The mean values, standard deviation for each functional pathway is reported.

| **No.** | **KEGG metabolism functions** | **Migration stages** | | | |
| --- | --- | --- | --- | --- | --- |
|  |  | **Autumn-H** | **Autumn-L** | **Spring-H** | **Spring-L** |
| a | Amino Acid Metabolism | 0.095±0.008 | 0.103±0.005 | 0.106±0.003 | 0.105±0.007 |
| b | Carbohydrate Metabolism | 0.103±0.005 | 0.108±0.007 | 0.093±0.010 | 0.087±0.012 |
| c | Energy Metabolism | 0.058±0.003 | 0.051±0.002 | 0.049±0.004 | 0.046±0.000 |
| d | Metabolism of Cofactors and Vitamins | 0.048±0.007 | 0.038±0.003 | 0.037±0.002 | 0.035±0.002 |
| e | Lipid Metabolism | 0.035±0.003 | 0.034±0.002 | 0.038±0.003 | 0.038±0.002 |
| f | Xenobiotics Biodegradation and Metabolism | 0.022±0.009 | 0.032±0.003 | 0.041±0.009 | 0.040±0.005 |
| g | Nucleotide Metabolism | 0.040±0.00 | 0.033±0.001 | 0.027±0.004 | 0.026±0.004 |
| h | Glycan Biosynthesis and Metabolism | 0.025±0.006 | 0.018±0.001 | 0.019±0.001 | 0.019±0.000 |
| i | Metabolism of Terpenoids and Polyketides | 0.019±0.001 | 0.019±0.000 | 0.020±0.002 | 0.020±0.000 |
| j | Enzyme Families | 0.019±0.001 | 0.019±0.000 | 0.018±0.001 | 0.017±0.000 |
| k | Metabolism of Other Amino Acids | 0.016±0.001 | 0.018±0.000 | 0.020±0.001 | 0.019±0.001 |
| l | Biosynthesis of Other Secondary Metabolites | 0.007±0.000 | 0.009±0.000 | 0.008±0.001 | 0.007±0.000 |

Table S8 Three-factor ANOVA of PICRUSTs2 function on predicting KEGG Level2 metabolic pathway in intestinal microbiota

| Abundance Rank | KEGG Level2 | Factors | *F* | *P* | Abundance Rank | KEGG Level2 | Factors | *F* | *P* |
| --- | --- | --- | --- | --- | --- | --- | --- | --- | --- |
| 1 | Amino acid metabolism | Season | 14.735 | 0.000*** | 7 | Nucleotide metabolism | Season | 87.024 | 0.000*** |
|  |  | Elevation | 4.566 | 0.037* |  |  | Elevation | 13.448 | 0.001** |
|  |  | Intestinal segments | 1.880 | 0.143 |  |  | Intestinal segments | 0.620 | 0.605 |
|  |  | Season $\times$Elevation | 9.546 | 0.003** |  |  | Season $\times$Elevation | 7.419 | 0.009** |
|  |  | Season$\times$Intestinal segments | 0.485 | 0.694 |  |  | Season$\times$Intestinal segments | 1.400 | 0.252 |
|  |  | Elevation$\times$Intestinal segments | 0.100 | 0.960 |  |  | Elevation$\times$Intestinal segments | 0.083 | 0.969 |
|  |  | Season$\times\mathrm{Elevation}\times$Intestinal segments | 0.120 | 0.948 |  |  | Season$\times\mathrm{Elevation}\times$Intestinal segments | 0.277 | 0.842 |
| 2 | Carbohydrate metabolism | Season | 43.895 | 0.000*** | 8 | Polysaccharide biosynthesis and metabolism | Season | 7.513 | 0.008** |
|  |  | Elevation | 0.042 | 0.839 |  |  | Elevation | 14.677 | 0.000*** |
|  |  | Intestinal segments | 0.833 | 0.481 |  |  | Intestinal segments | 0.288 | 0.834 |
|  |  | Season $\times$Elevation | 4.381 | 0.041* |  |  | Season $\times$Elevation | 17.724 | 0.000*** |
|  |  | Season$\times$Intestinal segments | 0.689 | 0.562 |  |  | Season$\times$Intestinal segments | 0.278 | 0.841 |
|  |  | Elevation$\times$Intestinal segments | 0.323 | 0.809 |  |  | Elevation$\times$Intestinal segments | 0.133 | 0.940 |
|  |  | Season$\times\mathrm{Elevation}\times$Intestinal segments | 0.283 | 0.838 |  |  | Season$\times\mathrm{Elevation}\times$Intestinal segments | 0.193 | 0.901 |
| 3 | Energy metabolism | Season | 87.301 | 0.000*** | 9 | Terpenoids and polyketides metabolism | Season | 8.032 | 0.006** |
|  |  | Elevation | 47.120 | 0.000*** |  |  | Elevation | 0.148 | 0.702 |
|  |  | Intestinal segments | 0.478 | 0.699 |  |  | Intestinal segments | 0.671 | 0.573 |
|  |  | Season $\times$Elevation | 6.346 | 0.015* |  |  | Season $\times$Elevation | 1.733 | 0.193 |
|  |  | Season$\times$Intestinal segments | 1.508 | 0.222 |  |  | Season$\times$Intestinal segments | 0.942 | 0.427 |
|  |  | Elevation$\times$Intestinal segments | 1.985 | 0.126 |  |  | Elevation$\times$Intestinal segments | 0.053 | 0.984 |
|  |  | Season$\times\mathrm{Elevation}\times$Intestinal segments | 0.725 | 0.541 |  |  | Season$\times\mathrm{Elevation}\times$Intestinal segments | 0.596 | 0.620 |
| 4 | Cofactor and vitamin metabolism | Season | 45.399 | 0.000*** | 10 | Enzyme Family | Season | 36.371 | 0.000*** |
|  |  | Elevation | 30.329 | 0.000*** |  |  | Elevation | 11.385 | 0.001** |
|  |  | Intestinal segments | 0.082 | 0.970 |  |  | Intestinal segments | 0.027 | 0.994 |
|  |  | Season $\times$Elevation | 13.291 | 0.001** |  |  | Season $\times$Elevation | 0.023 | 0.880 |
|  |  | Season$\times$Intestinal segments | 0.338 | 0.798 |  |  | Season$\times$Intestinal segments | 2.874 | 0.044* |
|  |  | Elevation$\times$Intestinal segments | 1.349 | 0.268 |  |  | Elevation$\times$Intestinal segments | 0.111 | 0.953 |
|  |  | Season$\times\mathrm{Elevation}\times$Intestinal segments | 0.660 | 0.580 |  |  | Season$\times\mathrm{Elevation}\times$Intestinal segments | 1.483 | 0.229 |
| 5 | Lipid metabolism | Season | 31.037 | 0.000*** | 11 | Other amino acid metabolism | Season | 41.195 | 0.000*** |
|  |  | Elevation | 0.957 | 0.332 |  |  | Elevation | 3.948 | 0.052 |
|  |  | Intestinal segments | 0.927 | 0.434 |  |  | Intestinal segments | 0.737 | 0.534 |
|  |  | Season $\times$Elevation | 0.471 | 0.496 |  |  | Season $\times$Elevation | 12.318 | 0.001** |
|  |  | Season$\times$Intestinal segments | 1.116 | 0.350 |  |  | Season$\times$Intestinal segments | 1.278 | 0.291 |
|  |  | Elevation$\times$Intestinal segments | 0.075 | 0.973 |  |  | Elevation$\times$Intestinal segments | 0.262 | 0.853 |
|  |  | Season$\times\mathrm{Elevation}\times$Intestinal segments | 0.436 | 0.728 |  |  | Season$\times\mathrm{Elevation}\times$Intestinal segments | 0.339 | 0.797 |
| 6 | Heterogeneous biomass metabolism and degradation | Season | 52.124 | 0.000*** | 12 | Other secondary metabolite biosynthesis | Season | 2.099 | 0.153 |
|  |  | Elevation | 5.379 | 0.024* |  |  | Elevation | 3.852 | 0.055 |
|  |  | Intestinal segments | 0.927 | 0.434 |  |  | Intestinal segments | 0.553 | 0.648 |
|  |  | Season $\times$Elevation | 9.091 | 0.004** |  |  | Season $\times$Elevation | 27.183 | 0.000*** |
|  |  | Season$\times$Intestinal segments | 1.489 | 0.227 |  |  | Season$\times$Intestinal segments | 0.768 | 0.517 |
|  |  | Elevation$\times$Intestinal segments | 0.122 | 0.947 |  |  | Elevation$\times$Intestinal segments | 0.574 | 0.634 |
|  |  | Season$\times\mathrm{Elevation}\times$Intestinal segments | 0.508 | 0.678 |  |  | Season$\times\mathrm{Elevation}\times$Intestinal segments | 0.610 | 0.611 |
